# Aerobic Metabolism in Vibrio cholerae Is Required for Population Expansion during Infection

**DOI:** 10.1128/mBio.01989-20

**Published:** 2020-09-01

**Authors:** Andrew J. Van Alst, Victor J. DiRita

**Affiliations:** a Department of Microbiology & Molecular Genetics, Michigan State University, East Lansing, Michigan, USA; Yale School of Medicine

**Keywords:** bacterial physiology, *in vivo* fitness, PDH, PFL, mucin metabolism, *Vibrio cholerae*, cholera

## Abstract

Vibrio cholerae replicates to high cell density in the human small intestine, leading to the diarrheal disease cholera. During infection, V. cholerae senses and responds to environmental signals that govern cellular responses. Spatial localization of V. cholerae within the intestine affects nutrient availability and metabolic pathways required for replicative success. Metabolic processes used by V. cholerae to reach such high cell densities are not fully known. We sought to better define the metabolic traits that contribute to high levels of V. cholerae during infection. By disrupting the pyruvate dehydrogenase (PDH) complex and pyruvate formate-lyase (PFL), we could differentiate aerobic and anaerobic metabolic pathway involvement in V. cholerae proliferation. We demonstrate that oxidative metabolism is a key contributor to the replicative success of V. cholerae *in vivo* using an infant mouse model in which PDH mutants were attenuated 100-fold relative to the wild type for colonization. Additionally, metabolism of host substrates, including mucin, was determined to support V. cholerae growth *in vitro* as a sole carbon source, primarily under aerobic growth conditions. Mucin likely contributes to population expansion during human infection as it is a ubiquitous source of carbohydrates. These data highlight oxidative metabolism as important in the intestinal environment and warrant further investigation of how oxygen and other host substrates shape the intestinal landscape that ultimately influences bacterial disease. We conclude from our results that oxidative metabolism of host substrates is a key driver of V. cholerae proliferation during infection, leading to the substantial bacterial burden exhibited in cholera patients.

## Abstract (precis)

Vibrio cholerae remains a challenge in the developing world and incidence of the disease it causes, cholera, is anticipated to increase with rising global temperatures and with emergent, highly infectious strains. At present, the underlying metabolic processes that support V. cholerae growth during infection are less well understood than specific virulence traits, such as production of a toxin or pilus. In this study, we determined that oxidative metabolism of host substrates such as mucin contribute significantly to V. cholerae population expansion *in vivo*. Identifying metabolic pathways critical for growth can provide avenues for controlling V. cholerae infection and the knowledge may be translatable to other pathogens of the gastrointestinal tract.

## INTRODUCTION

Vibrio cholerae causes the diarrheagenic disease cholera in humans and is particularly problematic in regions of the world with poor water sanitation. Ingesting contaminated water sources containing sufficiently high numbers of V. cholerae bacterial cells leads to infection characterized by excessive fluid loss and a substantial bacterial burden of V. cholerae during the acute phase of disease. In the human gastrointestinal tract, V. cholerae can proliferate to numbers as high as 10^6^ to 10^8^ cells per gram of stool (1). In this study, we sought to understand the metabolic requirements for V. cholerae that support such substantial population expansion within the gut.

The mucous lining of the gastrointestinal tract, which typically serves as a barrier to infection, is saturated with a variety of carbohydrates. Mucin is a glycoprotein and the primary macromolecule of mucus. Mucin consists of a protein backbone decorated with *O*-linked glycan chains containing sugars such as *N*-acetylgalactosamine, *N*-acetylglucosamine, galactose, fucose, and sialic acid (2). Commensal mucin-degrading bacteria, such as *Bacteroides* spp. and Akkermansia mucinophila, contain numerous mucinolytic enzymes capable of releasing these sugars from the mucin glycan chain to support growth (2, 3). Mucin degradation is also a feature of bacterial pathogens such as Shigella flexneri, Helicobacter pylori, and enterohemorrhagic Escherichia coli (4–7). V. cholerae contains a number of mucolytic glycosyl hydrolases that are predicted to release glycans from mucin polysaccharides (8–10). Indeed, previous studies have linked mucus carbohydrate metabolism with infection, as V. cholerae mutants defective for *N*-acetylglucosamine and sialic acid metabolism were attenuated for colonization in the infant mouse (9–11). The mechanism of acquisition of these mucin carbohydrates may be through both phosphoenolpyruvate phosphotransferase-dependent and independent systems to support growth *in vivo* (12). Although mucin can serve as a substrate for growth, chemical reduction of intestinal mucus during infection leads to increased numbers of V. cholerae, indicating that mucus also contributes to intestinal protection and clearance of the bacteria (13).

V. cholerae harbors the complete enzymatic pathways for the Embden-Meyerhof-Parnas (EMP/glycolysis) pathway, the Entner-Doudoroff (ED) pathway, and the pentose phosphate (PP) pathway (14, 15). The EMP and ED pathways predominantly generate the energy necessary for V. cholerae growth and proliferation. Additionally, previous work has shown that these pathways promote virulence factor production, although the direct cause for this effect is unknown (15, 16). In contrast, the PP pathway does not appear to play a significant role in the growth or colonization of V. cholerae during infection (17). These pathways culminate with the formation of pyruvate, which can then be used by the bacterium to fuel either aerobic or anaerobic metabolism to generate energy for the cell.

To expand our understanding of carbohydrate metabolism and its impact on V. cholerae *in vivo* fitness, we targeted the pyruvate dehydrogenase (PDH) complex and pyruvate formate-lyase (PFL), which both function to convert pyruvate to acetyl coenzyme A (acetyl-CoA) (18, 19). Examination of PDH and PFL mutants enables us to assess the contributions of aerobic and anaerobic metabolism to the expansion of V. cholerae during infection. The conversion of pyruvate to acetyl-CoA precedes the tricarboxylic acid (TCA) cycle, as the first step in the cycle requires acetyl-CoA to generate citrate. In previous work, V. cholerae mutants defective in the TCA cycle expressed increased levels of *toxT*, which encodes the major virulence gene activator in V. cholerae. This finding suggested a link between acetyl-CoA and virulence expression (20). However, these TCA cycle mutants were not tested *in vivo* and have been investigated only in classical biotype strains, not in strains of the El Tor biotype. Classical V. cholerae predominated among epidemic isolates prior to 1961, when it was supplanted by the El Tor biotype (21). The biotypes are differentiated by numerous physiological attributes that contributed to displacement of the classical biotype by the El Tor biotype (22–25). Some of these are encoded on genomic islands unique to the El Tor biotype that contribute to phage resistance or acquisition of substrates (26, 27).

In this study, we assessed pathways of carbohydrate metabolism as they contribute to growth, virulence factor production, and colonization of V. cholerae El Tor strain C6706. By targeting the PDH complex and PFL, we are able to draw conclusions about the aerobic and anaerobic metabolic processes that facilitate population expansion of V. cholerae during infection. Our results provide evidence supporting the importance of a functional PDH complex during infection, with significantly less reliance on PFL function. This indicates that oxidative metabolism primarily drives the growth and proliferation required to amass the high bacterial cell density observed during the disease cholera. The defects in colonization observed with strains lacking a functional PDH are attributable primarily to metabolic deficiencies, as virulence factor production was unaffected by mutation in these metabolic pathways. Given what is known in regard to oxygen availability within the intestinal environment, being highest in the intestinal crypts and decreasing to near hypoxia in the lumen, we can deduce the biogeographical localization of replicative V. cholerae (28). Our work suggests that replication within intestinal crypt spaces, observed by others (13), is enabled due to the higher oxygenation of this site than of the lumen. Furthermore, by using the physiologically relevant growth substrate mucin, we could closely reflect, and assess, the growth substrates typically encountered by V. cholerae during infection. The results of this study further our understanding of central metabolism and its contribution to V. cholerae infectivity and *in vivo* growth.

## RESULTS

### Transposon mutagenesis screen identified the pyruvate dehydrogenase complex as important for growth on mucin.

We hypothesized that intestinal mucin would serve as a growth substrate for V. cholerae during colonization. In a pilot experiment, V. cholerae was observed to exhibit enhanced growth in minimal medium supplemented with mucin (see Fig. S1 at https://doi.org/10.5281/zenodo.3966283). We then performed a transposon mutant library screen of V. cholerae El Tor strain C6706 on minimal medium supplemented with 0.5% mucin (Type III; Sigma). Genes encoding two of the three components of the pyruvate dehydrogenase (PDH) complex were identified in our screen, *aceE* (VC2414) and *aceF* (VC2413) (Table S1 at https://doi.org/10.5281/zenodo.3966283). The third component of the PDH complex, *lpdA* (VC2414), was also defective for growth in our screen; however, growth of this transposon mutant was also severely attenuated for growth on LB, as this enzyme also functions in the alpha-ketoglutarate dehydrogenase (AKGDH) and glycine cleavage multienzyme (GCV) systems (29). Because of its pleiotropic growth defect, we did not further investigate an *lpdA* mutant.

### The pyruvate dehydrogenase complex supports aerobic growth on mucin.

As a glycoprotein, mucin is coated in glycans, contributing to the protective function of the mucous barrier (30). To study mucin from a physiologically relevant site of infection, and to avoid potential contaminants in commercially purified mucin that may impact V. cholerae growth (31), we purified mucin from the small intestines of healthy adult pigs using a guanidine hydrochloride (GuHCl) extraction procedure. The purified mucin was then analyzed by high-performance anion-exchange chromatography coupled with pulsed amperometric detection (HPAEC-PAD; GlycoAnalytics) (32, 33). Mucin obtained by this method showed a high relative presence of the mucin carbohydrates galactosamine, glucosamine, galactose, fucose, and sialic acids Neu5Ac and Neu5Gc compared to nonmucin monosaccharides glucose and mannose (Table S2 at https://doi.org/10.5281/zenodo.3966283).

**FIG 1:**
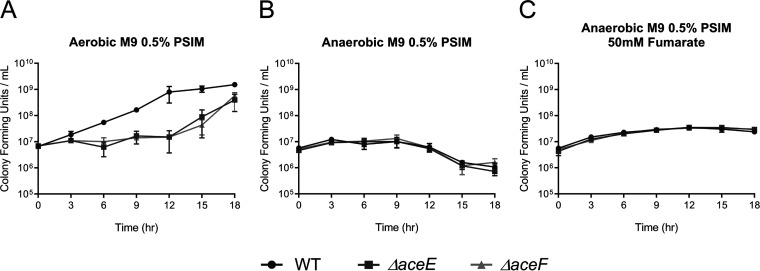
Growth curves of WT, *ΔaceE*, and *ΔaceF* strains in M9 minimal medium supplemented with 0.5% purified porcine small intestinal mucin (PSIM) grown aerobically (A), anaerobically (B), or anaerobically and supplemented with 50 mM fumarate (C). Data represent the averages and SEMs for three independent biological replicates.

When grown in M9 minimal salts medium supplemented with 0.5% purified small intestinal mucin (PSIM), isogenic *ΔaceE* and *ΔaceF* PDH mutants were defective for growth in aerobically grown cultures compared to the wild type (WT) (Fig. 1A). This phenotype was complemented for both the *ΔaceE* and *ΔaceF* mutants using the isopropyl β-d-1-thiogalactopyranoside (IPTG)-inducible pMMB66EH vector in M9 0.5% glucose medium (Fig. S2 at https://doi.org/10.5281/zenodo.3966283). To verify that the PDH complex does not contribute to anaerobic proliferation on mucin, we measured growth under anaerobic conditions. Under these conditions, PDH mutants grew comparably to the wild type (Fig. 1B). To determine if addition of an alternative electron acceptor would elicit a growth difference between the wild type and PDH mutants, 50 mM fumarate, which enhances V. cholerae growth via anaerobic respiration (34), was added to the growth medium. No growth disparity was observed between the wild type and PDH mutants with the addition of 50 mM fumarate (Fig. 1C). The observed phenotypes indicate that the PDH complex is not required for anaerobic growth. Energy generation under anaerobic conditions is likely due to an active pyruvate formate-lyase converting pyruvate to formate and acetyl-CoA for mixed acid fermentation (35) and acetolactate synthase, which converts pyruvate to (*S*)-2-acetolactate in the first step of 2,3-butanediol fermentation (25). The growth disparity observed in the minimal mucin medium under aerobic conditions was less pronounced in LB medium, which contains less than 100 μM collective sugars and primarily supports growth through amino acid catabolism (Fig. S3 at https://doi.org/10.5281/zenodo.3966283) (36). As a growth defect was observed in LB medium, we wanted to test whether this growth delay was attributable solely to perturbed carbohydrate metabolism or if growth on amino acids was also negatively impacted by a disrupted PDH complex. In M9 supplemented with 0.2% Casamino Acids under aerobic growth conditions, the *ΔaceE* and *ΔaceF* mutants did not to grow at all (Fig. S4 at https://doi.org/10.5281/zenodo.3966283). These findings suggest that aerobic amino acid catabolism may also have contributed to the phenotype illustrated in Fig. 1A, as mucin molecules contain, among other amino acids, proline, threonine, and serine in repeat glycan attachment moieties and have previously been shown to support V. cholerae growth *in vitro* (37). It is therefore unclear what component of LB supports growth of the *ΔaceE* and *ΔaceF* mutants.

### Pyruvate formate-lyase supports anaerobic growth on mucin.

**FIG 2:**
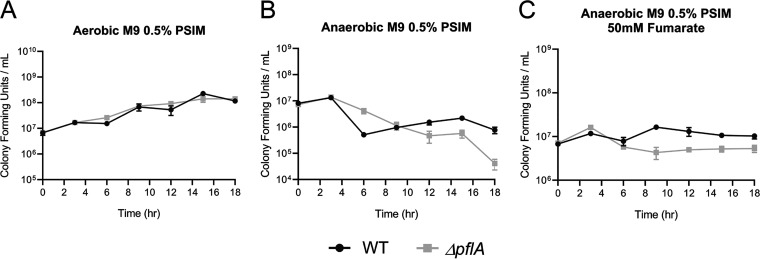
Growth curves of WT and *ΔpflA* strains in M9 minimal medium supplemented with 0.5% purified porcine small intestinal mucin grown aerobically (A), anaerobically (B), or anaerobically and supplemented with 50 mM fumarate (C). Data represent the averages and SEMs for three independent biological replicates.

As pyruvate formate-lyase (PFL) also converts pyruvate to acetyl-CoA, we sought to investigate the role of PFL in the catabolism of mucin. To accomplish this, an isogenic mutant strain with a deletion of *pflA* (VC1869) was tested for *in vitro* growth on PSIM. In M9 minimal salts medium supplemented with 0.5% PSIM, the *ΔpflA* mutant grew comparably to the wild type under aerobic growth conditions (Fig. 2A) and poorly compared to the wild type (which did not thrive itself) when cultured anaerobically, in both the absence and presence of 50 mM fumarate (Fig. 2B and C). This growth defect was complemented for *pflA* using an IPTG-inducible pMMB66EH vector in M9 0.5% glucose 50  mM fumarate medium (Fig. S5 at https://doi.org/10.5281/zenodo.3966283). The disparity in growth between the wild-type and *ΔpflA* strains under anaerobic growth conditions indicates that PFL can indeed generate energy from mucin during anaerobic growth. As PFL is expected to function primarily in the metabolism of carbohydrates, it was not surprising to see growth comparable to that of the wild type in LB medium, both aerobic and anaerobic, but it was intriguing to find that the *ΔpflA* mutant remained viable longer than the wild type in LB medium without the addition of 50 mM fumarate (Fig. S6 at https://doi.org/10.5281/zenodo.3966283).

### Cholera toxin production in PDH mutants is equivalent to that of the wild type under both standard and anaerobic toxin-inducing conditions.

**FIG 3:**
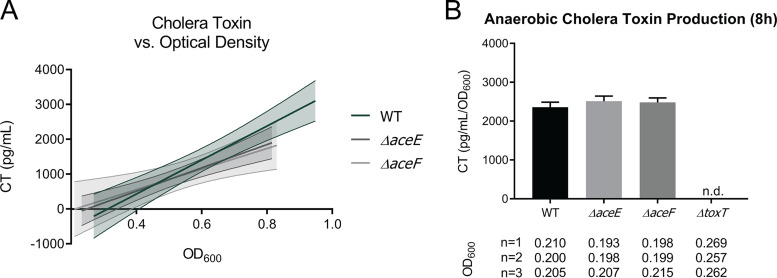
Cholera toxin (CT) production for WT, *ΔaceE*, *ΔaceF*, and *ΔtoxT* strains. (A) CT output as a function of optical density (OD_600_) under standard AKI toxin-inducing conditions. Data points were collected from three biological replicates, and a line of best fit with 95% confidence intervals was plotted. WT ODs higher than 0.9 were excluded to better superimpose with *ΔaceE* and *ΔaceF* strain OD values. A simple linear regression found no significant differences between WT, *ΔaceE*, and *ΔaceF* strain CT production. *ΔtoxT* control was not plotted because no toxin was detected. Statistical analysis was performed using GraphPad Prism. (B) CT values relative to optical density (pg/ml/OD_600_) under anaerobic AKI toxin-inducing conditions at the 8 h time point are reported. The optical densities for the biological replicates are displayed below the corresponding strain on the *x* axis in each graph. Data represent the averages and SEMs for three biological replicates.

Cholera toxin is the primary virulence determinant of V. cholerae. To determine whether the PDH complex influences production of cholera toxin, wild-type and PDH mutant strains were grown under conditions referred to as “AKI” to induce virulence factor production (38). Previous findings with strains of the classical biotype demonstrate that disruption of the TCA cycle increases *toxT* expression and suggest a link between acetyl-CoA and virulence expression (20). As the PDH complex is the primary enzyme responsible for the production of acetyl-CoA under aerobic growth conditions, we anticipated that mutants lacking it would produce cholera toxin levels below that of the wild type. However, we observed no significant difference in cholera toxin produced in the WT and PDH mutant strains under either standard or anaerobic AKI conditions. For standard AKI conditions, cholera toxin levels were measured as a function of optical density, with the *ΔtoxT* (VC0838) mutant included as a negative control, as ToxT stimulates cholera toxin production (Fig. 3A) (39). Overall, the wild type produced more total cholera toxin as it reached a higher final optical density than PDH mutants, yet when determining individual cellular capacities for cholera toxin production were determined, it was found that at similar optical densities, cholera toxin output in the PDH mutants was comparable to that in the wild type. Cholera toxin production levels at 4 h, 5 h, 6 h, 7 h, 8 h, and 24 h are provided in Fig. S7 at https://doi.org/10.5281/zenodo.3966283. Cholera toxin levels were also measured after 8 h and 20 h of growth under anaerobic AKI conditions and were again similar between the wild-type and PDH mutant strains (Fig. 3B and Fig. S7). These findings indicate that the PDH complex does not affect cholera toxin production in V. cholerae El Tor C6706.

### Functional PDH activity is not required for expression of *toxT*, *ctxA*, and *tcpA*.

**FIG 4:**
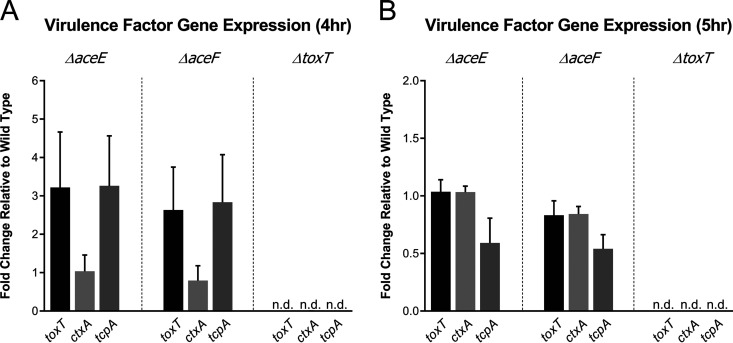
Relative fold change of *toxT*, *ctxA*, and *tcpA* transcript levels compared to wild-type expression. RNA was isolated from WT, *ΔaceE*, *ΔaceF*, and *ΔtoxT* cultures grown under standard AKI toxin-inducing conditions at 4 h and 5 h. Expression data were calculated by ΔΔ*C_T_* using *recA* as an internal control. Data represent the averages and SEMs for three independent biological replicates.

The relative expression of the master virulence regulator *toxT* and primary virulence factors *ctxA* and *tcpA* was determined by real-time quantitative PCR (RT-qPCR). The 4 h and 5 h time points of *in vitro* standard AKI conditions were selected to compare relative expression profiles, as *toxT* expression has been observed to be high at these time points (40). PDH mutant strains at 4 h exhibited somewhat elevated levels of *toxT* and *tcpA* and similar *ctxA* expression compared to those of the wild-type, with no transcripts detected in the *ΔtoxT* control, as expected (Fig. 4A). At 5 h, PDH mutant strains exhibited wild-type *toxT* and *ctxA* expression and a 2-fold reduction in *tcpA* expression (Fig. 4B). Although there was variability in the expression of these virulence genes at the given time points, we conclude that the PDH complex is not required for virulence gene expression.

### A functional pyruvate dehydrogenase complex is necessary for colonization of the infant mouse.

Based on our findings that V. cholerae PDH mutants are defective for aerobic carbohydrate metabolism, we sought to examine whether this pathway was required to support growth *in vivo*. The infant mouse model is used extensively to investigate intestinal colonization by V. cholerae (41). Infant mice produce a mucous layer in the intestine that can serve as a substrate for V. cholerae growth (13), although they also have reduced resident microbiota and a less developed immune system than adult mice (42). The reduction in resident flora increases the necessity for V. cholerae to liberate mucin glycans for substrate utilization, as the commensal population does not provide this resource as in other systems (43).

**FIG 5:**
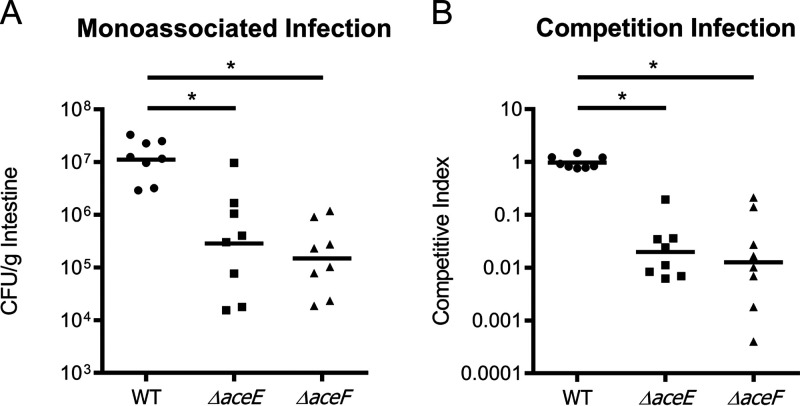
Infant mouse colonization assays of WT, *ΔaceE*, and *ΔaceF* strains after 20 h. (A) Monoassociated infections of 3- to 5-day-old infant mice. (B) Competition infections of 3- to 5-day-old infant mice. Competitive index scores were calculated as ratios of output versus input [(target_output_/*ΔlacZ*_output_)/(target_input_/*ΔlacZ*_input_)]. WT, *ΔaceE*, and *ΔaceF* strains were coinoculated with an *aceE*^+^*/aceF*^+^ *ΔlacZ* strain (PDH^+^) to determine the relative fitness of each test strain. Data for each experiment was obtained from eight independent mouse colonization infections in which the entire intestinal tract (small intestine, large intestine, and cecum) was extracted and homogenized for bacterial enumeration. Bars represent geometric means. Statistical analysis was performed using GraphPad Prism where significance was tested on log-transformed data by ANOVA with *post hoc* Tukey’s test. *, *P* < 0.05.

In this study, we orogastrically infected CD-1 mouse neonates with ∼10^6^ CFU bacterial cells to compare colonizations by wild-type and PDH mutant V. cholerae. In monoassociated infections, PDH mutants were attenuated for colonization approximately 100-fold compared to the wild type (Fig. 5A). We also assessed direct *in vivo* competition with wild type by coinfecting each mutant with a PDH^+^ *ΔlacZ* (VC2338) strain of V. cholerae. PDH mutant strains exhibited attenuation in competition with the wild type similar to that observed in monoassociated infections, with mutant recovery approximately 100-fold lower than that of the wild type after coinfection (Fig. 5B). These data indicate a requirement for a functionally active PDH complex to support colonization of the infant mouse, suggesting that oxidative metabolism of carbohydrate substrates is critical for colonization and population expansion.

**FIG 6:**
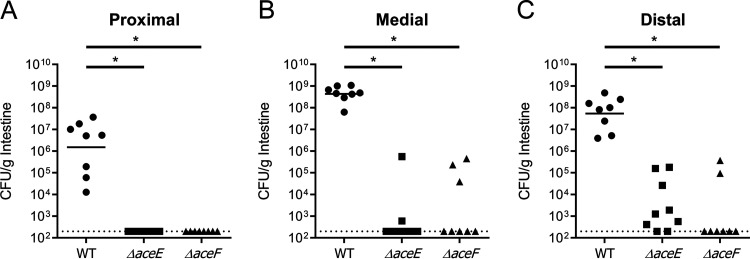
Infant mouse colonization of WT, *ΔaceE*, and *ΔaceF* strain monoassociated infections in the proximal (A), medial (B), and distal (C) portions of the small intestine after 20 h. Data for each segment were obtained from 8 independent mouse colonization infections. The bars represent the geometric means and could be plotted only for the WT strain. Statistical analysis was performed using GraphPad Prism where significance was tested on nontransformed data by Kruskal-Wallis analysis with *post hoc* Dunn’s test. *, *P* < 0.05.

As oxygen levels (44, 45) and mucin composition (46) fluctuate along the length of the small intestine, we investigated the relative importance of PDH function across the longitudinal axis in the infant mouse intestine. One-centimer-long pieces of intestine were harvested from the proximal, medial, and distal regions of the small intestine and assayed for recoverable CFU. Throughout the small intestine, V. cholerae PDH mutants were recovered at levels well below that of the wild type and in some cases were not detected, as counts were below our limit of detection (Fig. 6). Here we again conclude that the PDH complex promotes V. cholerae colonization and that oxidative metabolism of carbohydrates is a key feature of V. cholerae growth and proliferation along the entire length of the small intestine. Additionally, bacterial loads of the PDH mutants across the individual intestinal segments do not appear to reflect the CFU-per-gram counts obtained from analyzing the entire gastrointestinal tract in the previous monoassociated infection (Fig. 5A). This suggests that the majority of PDH mutants detected in the previous monoinfection experiment resided within either the cecum or large intestine, sites anticipated to support more anaerobic metabolism (45). This finding further supports the necessity for an active PDH, particularly at the primary site of infection in the small intestine.

### Pyruvate formate-lyase provides minor growth support during infection.

As aerobic metabolism was determined to be beneficial to population expansion of V. cholerae, we wanted to explore the contributing effects of anaerobic metabolism to colonization. Oxygen gradation within the small intestine maintains the highest oxygen availability in the intestinal crypts and nears hypoxia at the villus tip (28, 47). To determine if anaerobic proliferation also contributes to population expansion of V. cholerae during infection, potentially in the more anoxic lumen of the small intestine, CD-1 mice were infected with ∼10^6^ CFU of the *ΔpflA* mutant. To accurately assess the importance of PFL during infection, recovery of V. cholerae was performed for the small intestine separately from the large intestine. As the large bowel is inherently more anoxic, where PFL would be expected to function more readily, we focused more on assessing the role of PFL in the small intestine as a more clinically relevant site for human V. cholerae infection.

**FIG 7:**
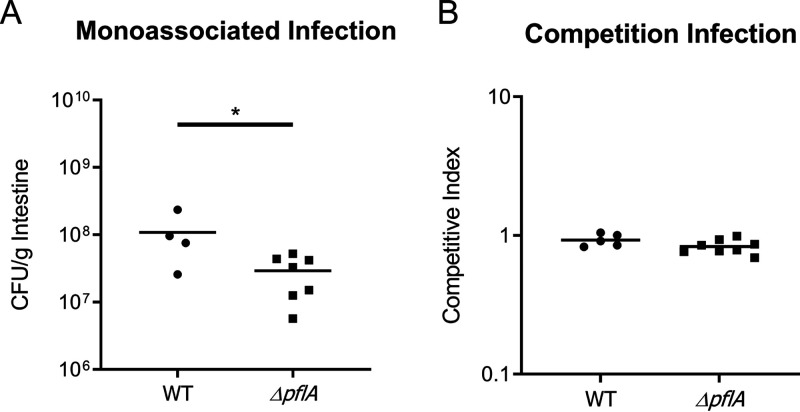
Infant mouse colonization assays of WT and *ΔpflA* strains in the small intestine after 20 h. (A) Monoassociated infections of 3- to 5-day-old infant mice. (B) Competition infections of 3- to 5-day-old infant mice. Competitive index scores were calculated as ratios of output versus input [(target_output_/*ΔlacZ*_output_)/(target_input_/*ΔlacZ*_input_)]. WT and *ΔpflA* strains were coinoculated with a *pflA*^+^ *ΔlacZ* strain to determine the relative fitness of each test strain. Data for each experiment was obtained from 4 or 5 independent mouse colonization infections for the WT and 7 or 8 mouse infections for the *ΔpflA* strain. The bar represents geometric mean. Statistical analysis was performed using GraphPad Prism where significance was tested on log-transformed data by Student’s *t* test. *, *P* < 0.05.

In monoassociated infections, the PFL mutant was attenuated for colonization approximately 2-fold compared to the wild type (Fig. 7A). These data suggest that PFL, and therefore anaerobic metabolism, provides a less critical level of energy production than PDH to support growth during infection. Our results are consistent with those of previous studies that investigated anaerobic nitrate respiration demonstrating a similar (2-fold) reduction in colonization of the infant mouse (48). However, in a competition experiment, the *ΔpflA* mutant colonized to levels equivalent to those of the wild type (Fig. 7B), essentially demonstrating no colonization defect at all. The same colonization pattern of the PFL mutant strain was observed in the large intestine (Fig. S8 at https://doi.org/10.5281/zenodo.3966283).

One hypothesis to explain this lack of fitness defect when coinfected with the wild type is that during coinfection, the *pflA*^+^ *ΔlacZ* strain may produce acetate, which can be metabolized by the *ΔpflA* mutant to mitigate the 2-fold defect seen in monoassociated infections (49). Acetate would provide acetyl-CoA by way of acetyl-CoA synthase 1 (ACS-1), circumventing the PDH/PFL carbohydrate utilization pathways (50). To test this hypothesis, we first demonstrated that all strains are capable of growth on acetate (Fig. S9 at https://doi.org/10.5281/zenodo.3966283). Then, to test whether metabolic rescue of the *ΔpflA* strain by *pflA*^+^ *ΔlacZ* occurs, monocultures and competition cultures were grown anaerobically in M9 minimal medium with 0.5% glucose. At both the 12 h and 20 h time points, the PFL mutant was found to be between 2- and 10-fold reduced compared to the *pflA*^+^ *ΔlacZ* strain for both monoculture and competition comparisons. These findings indicate that the similar output ratios detected in competition *in vivo* assays are unlikely related to acetate supplementation by the *pflA*^+^ *ΔlacZ* strain (Fig. S10 at https://doi.org/10.5281/zenodo.3966283).

## DISCUSSION

Oxygen-dependent metabolism is key to pathogenicity for many gastrointestinal microbes. Pathogens that actively manipulate the host environment to oxygenate the gut rapidly proliferate during infection. Although a direct link has yet to be determined, the cholera toxin of V. cholerae may increase oxygen availability in the gut through its influence on optimal TCA cycle activity during infection (51). In other gastrointestinal pathogens, oxidative metabolism is supported by inducing inflammation at the site of infection. Inflammation in response to Citrobacter rodentium or Salmonella enterica serovar Typhimurium infections promotes colonization, proliferation of the microbe, and disease as a result of increased aerobic metabolism (52, 53). While V. cholerae infection does not lead to significant changes in gross pathology of intestinal architecture and cholera is not typically characterized as a proinflammatory infection, inflammatory markers are increased in animal models and human infection (54, 55). Whether oxygen levels in the gut are elevated due to this innate immune response has yet to be determined. There is evidence to support inflammation promoting V. cholerae colonization in some circumstances. In V. cholerae strain V52, the type VI secretion system (T6SS) increases intestinal inflammation in the infant mouse and promotes increased colonization levels (56). Also, the newly emerged El Tor Haitian variant strain has higher virulence in animal models, reaching a higher bacterial cell burden than previously characterized strains and causing elevated inflammation and epithelial cell damage (57). Evolved V. cholerae strains equipped to withstand an inflamed environment could benefit from increased oxygen availability to generate more energy to support growth and proliferation.

Oxygen contribution to V. cholerae pathogenicity has been examined principally in regard to the ToxR/TcpP/ToxT virulence cascade. For example, the regulators AphB and OhrR respond to reduced environmental oxygen by activating *tcpP* expression (58). Further, decreased oxygen levels under stationary culture conditions stimulates ToxR-TcpP interaction (59) and activation of *toxT* transcription (40). Translating these *in vitro* results to the context of oxygen distribution *in vivo* would imply that the anoxic lumen primes V. cholerae for virulence gene expression prior to accessing the more oxygenated host epithelium. The radial oxygen gradient within the intestine would therefore influence optimal timing of V. cholerae virulence expression. In this work, we sought to explore how oxygen-dependent and independent metabolic pathways influence population expansion during infection, but not necessarily as they relate to virulence gene expression.

Oxygen availability in the crypt spaces of the intestine, along with the presence of carbohydrate-rich mucin molecules, could vastly improve growth and proliferation of V. cholerae at this site. The mucous lining of the gastrointestinal tract protects the host epithelium from both resident and transient microorganisms to maintain gut homeostasis (60). V. cholerae has the capacity to bypass this host defense through motility (61) as well as to exploit it for growth substrates. However, this does not suggest that the mucous layer is inconsequential to curtailing the effects of V. cholerae pathogenicity. In mice with a chemically degraded mucous layer, V. cholerae bacterial counts exceeded those of untreated mice (13), indicating that mucus contributes to abatement of disease. Similarly, *Muc2^−/−^* mice, which lack the primary secretory mucin of the intestinal tract, MUC2, exhibit inflammation in the large intestine due to commensal population interactions with the epithelium as well as exacerbated infection in Citrobacter rodentium and *Salmonella* Typhimurium challenge models (62–64). Thus, the mucus serves a protective function against V. cholerae yet is exploited to benefit the microbe.

The sequential progression of V. cholerae pathogenicity is tightly linked to bacterium-mucin interactions. Adherence to host mucin by GbpA, an *N*-acetylglucosamine binding protein, is a key step in colonizing the small intestine (65). Upon reaching the host epithelium, V. cholerae establishes an adherent microcolony (66). Within this microenvironment, mucin breakdown products and the presence of oxygen help drive the population expansion of V. cholerae (this work). Stimulation of the host epithelium by cholera toxin induces production and secretion of goblet cell mucin (67) in addition to other host-derived nutrients, such as iron and long-chain fatty acids (51) and potentially oxygen. As the population of cells rapidly expands, mucin breakdown products stimulate motility of V. cholerae (68), a trait required for optimal colonization of the proximal and medial portions of the small intestine (13, 69, 70). This interaction likely contributes to population dynamics observed for V. cholerae whereby it migrates counter to intestinal flow in the later stages of infection to populate the proximal and medial portions of the intestine (71). This motility response acts in coordination with the secreted mucolytic hemagglutinin/protease (HapA) of V. cholerae, used during cellular detachment (72, 73). HapA is stimulated both by high cell density through the activity of the regulator HapR and directly when in the presence of mucin (74). As V. cholerae exits the host, a fraction of the population is embedded in mucin (75), which may influence hyperinfectivity of human-passaged V. cholerae (76). From initial inoculation into the human gut to the eventual passaging of the bacteria, interactions between V. cholerae and mucus substantially influence V. cholerae pathogenicity.

Our work postulates that mucin metabolism enhances proliferation of V. cholerae during the course of infection. V. cholerae is a facultative anaerobe, and we sought to uncover whether aerobic or anaerobic metabolism enables it to grow to high levels during infection. We assessed the *in vivo* fitness of strains lacking either the *aceE-* or *aceF*-encoded components of the pyruvate dehydrogenase (PDH) complex or *pflA-*encoded pyruvate formate-lyase (PFL). These enzymes catalyze production of acetyl-CoA from pyruvate either aerobically (PDH) or anaerobically (PFL). In Escherichia coli, PFL is induced only during anaerobiosis, whereas the PDH complex can function in both anaerobic and anaerobic environments (77, 78). However, unlike what is observed in E. coli, in our work, V. cholerae lacking PFL was not rescued for anaerobic growth to any noticeable extent by having a functional PDH. This enabled us to differentiate the contribution of aerobic and anaerobic metabolism by investigating PDH and PFL mutants. The significant loss of fitness by the *ΔaceE* and *ΔaceF* strains compared to the wild-type strain suggests that V. cholerae population expansion in the small intestine is driven largely by aerobic, oxidative metabolism. This is consistent with V. cholerae preferentially localizing to the epithelial crypts (13), with greater oxygenation that enables oxidative metabolic pathways to generate energy (28). The radial distribution of oxygen in the intestine therefore biogeographically relegates replicative V. cholerae cells primarily to the epithelium, as opposed to the more anoxic lumen. Our results do not completely rule out some anaerobic growth and expansion of V. cholerae during infection, as the *ΔpflA* mutant colonized to levels about half those of the wild type, similar to what is observed with other anaerobic metabolism-deficient strains (25, 34, 48). Additionally, there is recent evidence to suggest that multiple anaerobic metabolic pathways function in tandem to support growth under anoxic conditions. A double mutant in ethanol fermentation and nitrate respiration showed a significant reduction in colonization, in contrast to single mutants that showed near-wild-type levels of colonization (79).

While expansion of V. cholerae *in vivo* evidently proceeds primarily through aerobic production of acetyl-CoA using PDH as opposed to anaerobic acetyl-CoA production using PFL, how it uses its reducing equivalents to generate energy from the electron transport chain is less certain from our work. Oxygen as a terminal electron acceptor is certainly possible given the availability of oxygen within the crypt epithelium and the presence of four terminal oxidase complexes in the V. cholerae genome (80), which will be the subject of future investigation. Previous *in vivo* transposon mutagenesis studies indicate that terminal oxidase function supports colonization, in particular a high-affinity cbb3 oxidase (81, 82). V. cholerae also maintains nitrate, fumarate, trimethylamine N-oxide (TMAO), and dimethyl sulfoxide (DMSO) reductases that can function as terminal electron acceptors in anaerobic respiration (80). Fumarate and TMAO support V. cholerae growth under anaerobic conditions (34), as does nitrate when in an alkaline environment (48), albeit not to the extent observed for oxidative growth. This is primarily due to the relatively low redox potentials of fumarate and TMAO relative to O_2_ and V. cholerae requiring alkaline pH environments for nitrate respiration, as it lacks a nitrite reductase needed to eliminate this toxic compound (48). DMSO, on the other hand, was not shown to support V. cholerae growth at all (34). However, growth *in vivo* with addition of the alternative electron acceptor TMAO induces high levels of cholera toxin (34). Infant mice infected with an inoculum of El Tor strain N16961 mixed with TMAO exhibited more severe signs of infection, suggesting a TMAO-dependent toxin production effect. Although anaerobic terminal reductases may not be the principal mode of V. cholerae growth and expansion *in vivo*, they are still likely to contribute to V. cholerae pathogenesis. Resolution of the role of different terminal reductases regarding growth and pathogenicity *in vivo* also awaits future examination by investigating V. cholerae terminal reductase mutants.

## MATERIALS AND METHODS

### Transposon mutagenesis library screen.

We used a nonredundant transposon mutant library collection constructed in the El Tor C6706 background (83). Using a 96-well plate replicator, the library was replica plated onto large LB kanamycin (0.05 mg/ml) agar plates and incubated overnight at 37°C. Subsequently, this LB-grown library was replica plated onto minimal MCLMAN medium (84) plates supplemented with 0.5% type III porcine gastric mucin (Sigma). Transposon insertion mutants that were qualitatively defective for growth compared to neighboring transposon insertion mutants were marked as deficient for mucin utilization for further investigation. The complete list of identified mutants is in Table S1 at https://doi.org/10.5281/zenodo.3966283.

### Porcine small intestinal mucus collection and mucin purification.

Fresh porcine small intestinal segments were harvested from healthy adult pigs from the Michigan State University Meat Lab. Mucus was scraped from the intestinal segments and purified in a manner similar to that previously described (85). Briefly, crude mucus was solubilized and resuspended in extraction guanidine hydrochloride (extraction GuHCl) (6 M guanidine hydrochloride, 5 mM EDTA, 0.01 M NaH_2_PO_4_ [pH 6.5]) and homogenized using a Dounce homogenizer. The crude mucus was then rocked overnight at 4°C, followed by centrifugation at 14,000 rpm and 10°C for 45 min. The supernatant was removed, and samples were washed again with extraction GuHCl. Samples were washed and centrifuged a total of five times or until the supernatant appeared clear for two consecutive washes. Mucin was then solubilized using 20 ml of reduction guanidine hydrochloride (6 M guanidine hydrochloride, 0.1 M Tris, 0.5 mM EDTA [pH 8.0]) with the addition of 25 mM dithiothreitol (DTT) as a powder just before use and rocked for 5 h at 37°C. A 75 mM concentration of iodoacetamide was added after incubation as a powder, and samples were rotated in the dark overnight. Samples were then centrifuged at 4,000 rpm for 45 min at 4°C. The supernatant was added to dialysis tubing and dialyzed in double-distilled water (ddH_2_O) for a total of six changes. The samples were flash-frozen using liquid nitrogen and lyophilized for purified mucin powder.

### Bacterial strains and growth conditions.

Vibrio cholerae and Escherichia coli strains used in this study are listed in Table S3 at https://doi.org/10.5281/zenodo.3966283. Unless otherwise specified, V. cholerae and E. coli strains were grown aerobically at 37°C on LB agar plates or with shaking at 210 rpm in LB broth. Where indicated, antibiotics were routinely added to the media at the following concentrations: 0.1 mg/ml of streptomycin, 0.1 mg/ml of ampicillin, and 0.05 mg/ml of kanamycin. LB medium was prepared according to a previously reported recipe (86); however, solid medium was made with a 1.5% (wt/vol) concentration of agar.

### Primers.

Primers used in this study are listed in Table S4 at https://doi.org/10.5281/zenodo.3966283.

### Plasmid construction.

Plasmid construct inserts were generated by PCR using Phusion high-fidelity polymerase (Thermo Scientific). Vector backbones were generated by plasmid purification using a Qiagen miniprep kit and subsequent restriction digestion.

A modified pKAS32 suicide vector was constructed to generate *ΔaceE*, *ΔaceF*, and *ΔpflA* strains (87). Primer sets were used to amplify 1,000-bp homologous regions upstream and downstream of the target gene. The pKAS32 vector was restriction digested using SacI and XbaI at 37°C for 1 h, followed by an additional 30 min at 37°C with alkaline phosphatase from calf intestine (CIP; New England BioLabs). Vector backbone and upstream and downstream segments were joined using Gibson assembly (New England BioLabs) and subsequently electroporated into electrocompetent E. coli S17 λpir and recovered on agar plates with LB ampicillin (0.1 mg/ml).

A description of the construction of complementation plasmids can be found in the supplemental methods at https://doi.org/10.5281/zenodo.3966283.

### Vibrio cholerae mutant construction.

Wild-type V. cholerae and E. coli strains were mated on LB agar plates at 37°C overnight. The mating was then plated on LB ampicillin (0.1 mg/ml) and polymyxin B (25 U/ml). Colonies were then subjected to streptomycin counterselection as described previously using counterselection plates with LB streptomycin (2.5 mg/ml) (87). Colonies were screened for the deletion using primer sets upstream and downstream of the pKAS32 homology regions.

A description of the generation of complementation strains can be found in the supplemental methods at https://doi.org/10.5281/zenodo.3966283.

### Growth curves.

M9 0.5% purified porcine small intestinal mucin (PSIM) was made by combing in a 1:1 mixture 2× M9 minimal medium and 2× (1%) PSIM prepared as a final 20-ml volume which was then autoclaved for 20 min at 121°C.

Strains were initially grown on plates with LB streptomycin (0.1 mg/ml) overnight at 37°C, and a single colony isolate was used to start a fresh broth culture on LB streptomycin (0.1 mg/ml) grown overnight at 210 rpm and 37°C. Overnight cultures were washed twice in phosphate-buffered saline (PBS) and resuspended to an optical density at 600 nm (OD_600_) of 1.0.

**(i) Aerobic growth curves.** For LB growth curves, a 1:1,000 dilution of a culture at an OD_600_ of 1.0 was used to inoculate prepared medium (either 2 ml or 50 ml, depending on the experiment) which was grown at 210 rpm and 37°C. For M9 plus 0.5% PSIM, 2 ml of medium was added to a 15-ml round-bottom tube and inoculated 1:250 with a culture at an OD_600_ of 1.0 and grown at 210 rpm and 37°C. At each time point, 100 μl was removed for dilution series plating.

**(ii) Anaerobic growth curves.** Anaerobiosis was achieved using a Coy anaerobic chamber. For LB growth curves, a 1:1,000 dilution of a culture at an OD_600_ of 1.0 was used to inoculate prepared medium and grown statically at 37°C. WT, *ΔaceE*, and *ΔaceF* strain growth curves were carried out in 50 ml of LB medium in a 125-ml flask, whereas later WT and *ΔpflA* strain LB growth curves were carried out in 2 ml of LB medium in a 15-ml round-bottom tube. For M9 0.5% PSIM, 2 ml of medium was added to a 15-ml round-bottom tube and inoculated 1:250 with a culture at an OD_600_ of 1.0 which was grown statically at 37°C. At each time point, the flask and tubes were swirled or vortexed and 100 μl was removed for dilution series plating. For growth curves including 50 mM fumarate, sodium fumarate (Sigma) reagent was used.

**(iii) Complementation growth curves.** Methods for complementation growth curves can be found in the supplemental methods at https://doi.org/10.5281/zenodo.3966283.

### AKI virulence-inducing conditions.

**(i) Standard AKI conditions.** Wild-type, *ΔaceE*, *ΔaceF*, and *ΔtoxT* strains were grown statically in 50 ml of prewarmed AKI medium in 50-ml conical tubes for 4 h at 37°C, followed by a transfer to 125-ml flasks and shaking at 210 rpm and 37°C (38). One milliliter of medium was removed at each time point and centrifuged at 14,000 rpm for 1 min. Supernatant was separated from the pellet and stored at −80°C for cholera toxin quantification. The bacterial pellet was resuspended in 1 ml of TRIzol (Ambion Life Technologies) and stored at −80°C for RNA isolation.

**(ii) Anaerobic AKI conditions.** Wild-type, *ΔaceE*, *ΔaceF*, and *ΔtoxT* strains were grown in 50 ml of prewarmed oxygen-depleted AKI medium in 50-ml conical tubes statically under anaerobic conditions using a Coy anaerobic chamber (88). One milliliter of medium was removed at each time point and centrifuged at 14,000 rpm for 1 min. The supernatant was separated from the pellet and stored at –80°C for cholera toxin quantification.

### Cholera toxin quantification by ELISA.

Cholera toxin in V. cholerae supernatants from standard and anaerobic AKI conditions was quantified by GM1 enzyme-linked immunosorbent assay (ELISA) as previously described (89, 90). GM1-coated microtiter plates were incubated with a 1:20 dilution of culture supernatant and detected using primary anti-cholera toxin and secondary horseradish peroxidase (HRP)-conjugated goat anti-rabbit IgG (Invitrogen). 1-Step Ultra TMB-ELISA (Thermo Scientific) reagent was added and stabilized using 2 M sulfuric acid. Colorimetric measurements were read at 450 nm, and the toxin concentration was determined by comparison to a standard curve using purified cholera toxin.

### RNA isolation and real-time quantitative PCR (RT-qPCR).

RNA was harvested from AKI toxin-inducing culture pellets preserved in 1 ml of TRIzol using an RNEasy kit (Qiagen) using on-column DNase digestion (Qiagen) followed by Turbo DNase digestion (Invitrogen). RNA concentration and quality were measured with a UV/VIS Spectrophotometer and visualized on a 2% agarose gel.

cDNA was generated from RNA using Superscript III reverse transcriptase (Thermo Scientific). RT-qPCRs were carried out using SYBR green master mix (Applied Biosystems) with 5 ng of cDNA. Primers used to detect *recA*, *toxT*, *ctxA*, and *tcpA* transcripts are listed in Table S4 at https://doi.org/10.5281/zenodo.3966283. Threshold cycle (ΔΔ*C_T_*) values were calculated using *recA* as the gene of reference (91).

### Infant mouse colonization assays.

All animal experiments in this study were approved by the Institutional Animal Care and Use Committee at Michigan State University.

Infant mice were infected as described previously (92). Three- to five-day-old CD-1 mice (Charles River, Wilmington, MA) were orogastrically inoculated with approximately 10^6^ bacterial cells 2 h after separation from the dam and maintained at 30°C. Mice were euthanized approximately 20 h after inoculation. Mouse intestinal segments were weighed and homogenized in 3 ml of PBS. Intestinal homogenates were serially diluted and plated on LB streptomycin (0.1 mg/ml) for monoassociated infections and LB streptomycin (0.1 mg/ml) and 5-bromo-4-chloro-3-indolyl-β-D-galactopyranoside (X-Gal) (0.08 mg/ml) for competition infections. Competition infections consisted of a 1:1 mixture of target strains with a *ΔlacZ* strain for differentiation by blue-white screening.

For PDH monoassociated and competition infections, the entire intestinal tract was homogenized for bacterial enumeration. In intestinal segment measurements, approximately 1 cm of intestine from each section (proximal, medial, and distal) was homogenized for bacterial enumeration between segments. For PFL monoassociated and competition infections, the intestinal tract was divided into small intestine and large intestine plus cecum. The divided intestinal portions were then homogenized for bacterial enumeration.

### Statistical methods.

For determining the relationship between cholera toxin output versus optical density among WT, *ΔaceE*, and *ΔaceF* strains, a simple linear regression and subsequent slope and intercept analysis were performed using GraphPad Prism software, which follows a method equivalent to analysis of covariance (ANCOVA).

For *in vivo* experiments, CFU-per-gram intestine and competitive index scores were log_10_ transformed and tested for normality using a Shapiro-Wilks test. Normally distributed data were then analyzed using either parametric Student’s *t* test or analysis of variance (ANOVA) with *post hoc* Tukey’s test to test for significance.

For *in vivo* intestinal segment data where bacterial loads were below the limit of detection, a nonparametric Kruskal-Wallis one-way analysis of variance was used with *post hoc* Dunn’s test to test for significance.
